# A dynamic online nomogram for predicting renal outcomes of idiopathic membranous nephropathy

**DOI:** 10.1186/s12911-024-02568-2

**Published:** 2024-06-19

**Authors:** Feng Wang, Jiayi Xu, Fumei Wang, Xu Yang, Yang Xia, Hongli Zhou, Na Yi, Congcong Jiao, Xuesong Su, Beiru Zhang, Hua Zhou, Yanqiu Wang

**Affiliations:** 1 Department of Nephrology, Shengjing Hospital of China Medical University, Shenyang, Liaoning People’s Republic of China; 2 Department of Clinical Epidemiology, Shengjing Hospital of China Medical University, Shenyang, Liaoning People’s Republic of China; 3 Department of Nephrology, The First Affiliated Hospital of Jinzhou Medical University, Jinzhou, Liaoning People’s Republic of China; 4 Department of Nephrology, The General Hospital of Angang Group, Anshan, Liaoning People’s Republic of China

**Keywords:** Idiopathic membranous nephropathy, Prognosis, Treatment response, Dynamic nomogram

## Abstract

**Background:**

Because spontaneous remission is common in IMN, and there are adverse effects of immunosuppressive therapy, it is important to assess the risk of progressive loss of renal function before deciding whether and when to initiate immunosuppressive therapy. Therefore, this study aimed to establish a risk prediction model to predict patient prognosis and treatment response to help clinicians evaluate patient prognosis and decide on the best treatment regimen.

**Methods:**

From September 2019 to December 2020, a total of 232 newly diagnosed IMN patients from three hospitals in Liaoning Province were enrolled. Logistic regression analysis selected the risk factors affecting the prognosis, and a dynamic online nomogram prognostic model was constructed based on extreme gradient boost, random forest, logistic regression machine learning algorithms. Receiver operating characteristic and calibration curves and decision curve analysis were utilized to assess the performance and clinical utility of the developed model.

**Results:**

A total of 130 patients were in the training cohort and 102 patients in the validation cohort. Logistic regression analysis identified four risk factors: course ≥ 6 months, UTP, D-dimer and sPLA2R-Ab. The random forest algorithm showed the best performance with the highest AUROC (0.869). The nomogram had excellent discrimination ability, calibration ability and clinical practicability in both the training cohort and the validation cohort.

**Conclusions:**

The dynamic online nomogram model can effectively assess the prognosis and treatment response of IMN patients. This will help clinicians assess the patient’s prognosis more accurately, communicate with the patient in advance, and jointly select the most appropriate treatment plan.

## Background

Idiopathic membranous nephropathy (IMN) is the most common pathologic type of adult nephrotic syndrome (NS) [1]. IMN is common in the middle-aged and elderly, the incidence has gradually increased in China in recent years, and there is a trend of younger age [2]. IMN is the second or third primary glomerulonephritis leading cause of end-stage renal disease (ESRD) in the USA and Europe [3], and approximately one third of IMN patients have a progressive disease course. The most frightening long-term consequence of IMN is progressive loss of kidney function. And among 60% of untreated patients, there are about 35% patients eventually develop to ESRD within 10 years [4–7].

In recent decades, some clinical [8, 9], pathological [10, 11], and genetic [12, 13] parameters have been identified as biomarkers for predicting the prognosis of IMN, and the Kidney Disease Improving Global Outcomes (KDIGO) 2021 clinical practice guideline have taken 24 h urinary protein (UTP), estimate glomerular filtration rate(eGFR), serum anti-phospholipase A2 receptor antibody (sPLA2R-Ab), serum albumin (ALB) and others as indicators that may be used to divide patients into categories of low, moderate, high, and very high risk of progressive loss of kidney function [14]. However, there are often inconsistencies in various indicators in clinical practice, such as high level proteinuria in patients with low titer sPLA2R-Ab, or high level proteinuria with nomal serum ALB. And there is currently no model that combines all of these clinical considerations. Therefore, there is an urgent need to develop a model that takes into account clinical factors, and use the cut-off value of the model to identify high-risk patients with poor prognosis, which is conducive to clinical application. In addition, if a patient is initiated immunosuppressive therapy according to the KDIGO 2021 clinical practice guideline, but the outcome of treatment is unclear. If a model can be used to predict patient outcomes and response to treatment, the model may help clinicians assess patient prognosis in advance, and can fully discuss with patients to determine the best treatment options to maximize the benefit of patients.

The nomogram is a useful and accessible tool for physicians to predict the disease progression, to plan for individualized treatment, and to decide the interval for follow-up [15, 16]. Nomograms have been previously developed for IMN [17–20], but most of the nomograms lack of external validity [17, 19], and no dynamic online nomogram related to IMN prognosis is found at present to our knowledge. Machine learning has recently been used to produce a prediction model for practice. Machine learning can help model information based on statistics, potentially revealing hidden dependencies between predictors and diseases. Previous studies have shown that machine learning algorithms such as extreme gradient boost (XGBoost), random forest (RF) and logistic regression (LR) have been used to predict or identify kidney disease [21, 22]. The purpose of this study is to establish a dynamic online nomogram model based on machine learning model, in order to accurately identify the prognosis and treatment response of IMN patients, and to help clinicians formulate personalized treatment plans.

## Methods

### Study cohorts

**Fig. 1:**
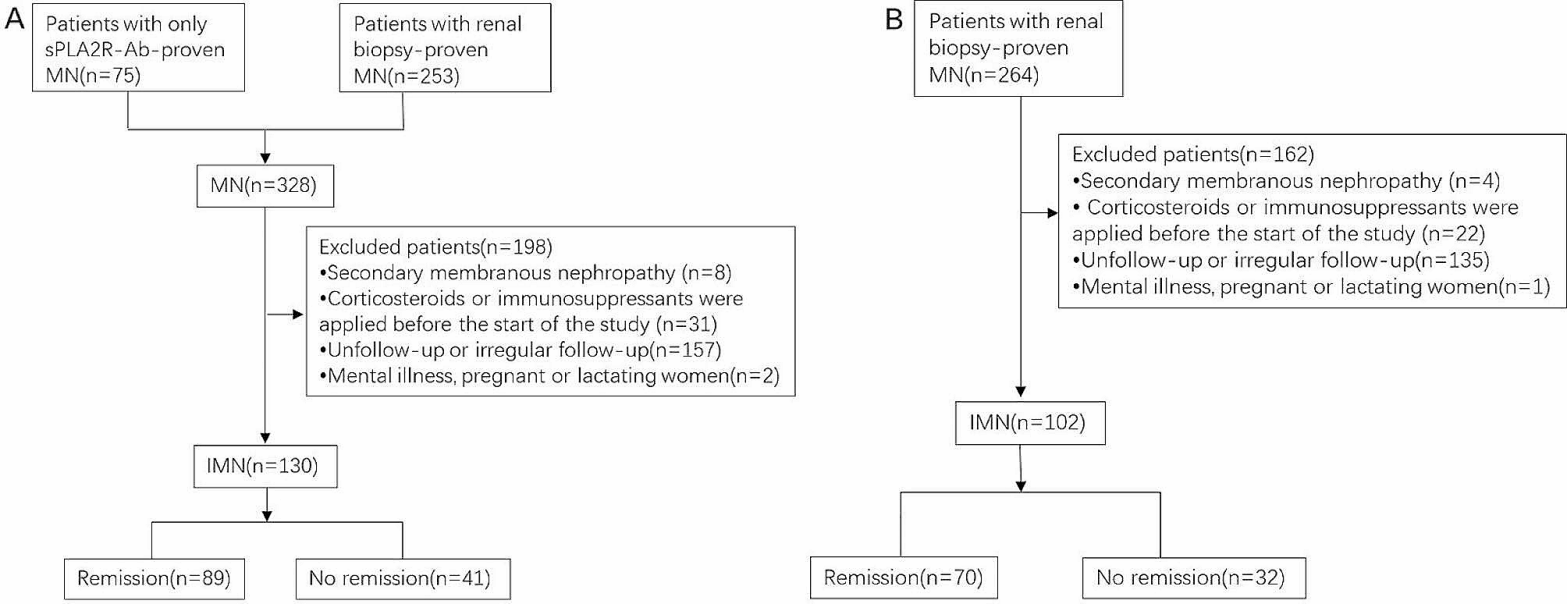
Flowchart of inclusion and exclusion in the training and validation cohort. **A**: In the training cohort; **B**: In the validation cohort

This was a retrospective analysis of multicenter study in 3 hospitals in Liaoning Province, northeast of China, which included 232 cases from September 2019 to December 2020. The training cohort included 130 patients from Shengjing Hospital of China Medical University, and the validation cohort was 102 patients from the First Affiliated Hospital of Jinzhou Medical University and the General Hospital of Angang Group (Fig. 1). The inclusion criteria for IMN were as follows: (i) patients with MN diagnosed by renal biopsy or a positive anti-PLA2R antibody test with NS. (ii) age 18 to 75 years. (iii) a follow-up time ≥ 24 months and with complete data which was obtained in our institution. The exclusion criteria were as follows: (i) secondary membranous nephropathy (SMN), including those with autoimmune disease, infection, malignancy, drug and heavy metal poisoning related MN. (ii) corticosteroids or immunosuppressants were applied before the start of the study. (iii) follow-up periods less than 24 months, or with missing data. iiii. patients with serious mental illness that is difficult to cooperate with treatment, and pregnant or lactating women. This study had been approved by the Ethics Committee of Shengjing Hospital affiliated to China Medical University, and informed consent was waived because it was a retrospective study(the ethics number: 2023PS847K).

### Clinical data collection

The baseline and follow-up data were extracted from patients’ records in hospital’s electronic medical system, including demographic characteristics, clinical variables, laboratory results. According to the manufacturer’s recommendation, detection of sPLA2R-Ab titer was performed using ELISA (E200908BU, Euroimmun, Germany), and a value ≥ 20 RU/ml was considered as positive. Renal biopsy was performed, and the biopsy sample examined by light microscopy, immunofluorescence, and electron microscopy. Membranous lesions from IMN cases were classified into four stages based on the criteria of Ehrenreich and Churg [23].

### Treatment options

The treatment strategy was based on the KDIGO 2021 clinical practice guideline [14]. Renin-angiotensin-aldosterone system (RAAS) inhibitors consist primarily of angiotensin-converting enzyme inhibitor (ACEi)/angiotensin-II receptor blocker (ARB). Immunosuppressant therapy includes cyclophosphamide (CTX) or calcineurin inhibitor (CNI). Targeted therapy refers to CD20 monoclonal antibody therapy, mainly including rituximab and obinutuzumab. Other immunosuppressant treatments include mycophenolate mofetil, leflunomide, tripterygium wilfordii and others.

### Outcome

The clinical endpoint was non-remission of proteinuria at 24 months. Complete remission (CR) was defined as achieving a normal level of proteinuria excretion of no more than 0.3 g per 24 h and with a stable eGFR. Partial remission (PR) was defined as proteinuria between 0.3 g and 3.5 g per 24 h, or a reduction in proteinuria of at least 50% compared with baseline and with a stable eGFR [24, 25]. Patients who did not meet any of those criteria were categorized as non-remission (NR). A stable eGFR was defined as an eGFR that remained unchanged or declined less than 15% during the period of follow-up.

### Model construction and performance evaluation

In the training cohort, we used univariate’ and multivariate’ logistic regression to screen for major risk factors with non-remission urine protein based on the patient’s baseline measurements, and constructed a nomogram based on XGBoost, RF, LR machine learning algorithms. Five key metrics are used to assess the effectiveness of the model: area under the receiver operating characteristic (AUROC), sensitivity, specificity, accuracy and F1-score.

### Internal and external validation of the model

The nomogram was subjected to 1000 bootstrap resamples for internal validation to assess its predictive accuracy, and was performed by a visual calibration plot. The discriminative ability of the model was determined by AUROC, which ranges from 0.5 to 1, and the AUROC was compared using Z test. Finally, to estimate the clinical utility of the model, the decision curve analysis (DCA) was performed by calculating the net benefits for a range of threshold probability. The external validity of the model was evaluated by the AUROC, calibration and decision curve analysis in an independent cohort.

### Statistical analysis

All the statistical analyses were done by SPSS26.0 and R 4.2.1. Normally distributed continuous variables were expressed with their means and standard deviations whereas non-normal continuous variables were expressed by their medians and interquartile ranges (IQR). Categorical variables were expressed with frequencies and percentages. The statistical significance between two cohorts was determined by T test or the Wilcoxon rank sum test for continuous variables and Chi-square test for categorical variables. Results with *P*<0.05 were considered statistically significant.

## Results

### Patient characteristics

232 IMN patients were enrolled in this study, and the characteristics were presented in Table 1. The training cohort included 85 males, and with the median age being 48 years and the median proteinuria was 7.0 g/d. The characteristics were compared between the training and validation cohorts, and it showed that there were significant differences in age, uric red blood cell (URBC), ALB, serum creatinine (Scr), blood urea nitrogen (BUN), and total cholesterol (TCHO). In the training cohort, 26 patients did not undergo renal biopsy due to personal willingness or physical condition, while the majority of patients with renal biopsy presented in stages 2 and 3. The patient’s treatment plan was based on the KDIGO 2021 clinical practice guideline, more than 60% of patients received RAAS inhibitors or immunosuppressant therapy in the training or validation cohort, and there were no significant differences in treatment regimens between the two groups. The follow-up time was 24 months, and the incidence of the endpoint of the IMN progression was 31.5% and 31.4% in the training and validation cohorts. In addition, we retrospectively analyzed the adjustment of the treatment regimen during the follow-up of the patients in the training cohort, it was found that 30 patients did not achieve remission even after changing the immunosuppressant regimen, and 7 of them made two adjustments to the treatment regimen and still had persistent urine protein.

**Table 1:** Characteristics of the overall population in the training and validation cohorts

Characteristic	Training cohort (*n* = 130)	Validation cohort (*n* = 102)	*P* Value
Man(%)	85(65.4)	75(73.5)	0.183
Age(year)	48.0 (39.0-56.3)	52.5 (45.0–60.0)	0.003
Weight(/kg)	73.0 (63.0-83.5)	75.0 (65.8–84.0)	0.871
Course ≥ 6 months(%)	38(29.2)	27(26.5)	0.642
History of hypertension(%)	35(26.9)	30(29.4)	0.675
URBC(/Hp)	7.3(2.8–16.0)	11.7(5.0-24.3)	0.001
UWBC(/Hp)	2.4(1.2–5.4)	3.1(1.6–5.2)	0.251
UTP(g/24 h)	7.0(4.5–10.7)	6.1(3.5–10.5)	0.132
HGB(g/L)	140.5(128.0-149.3)	143.5(132.0-155.3)	0.101
ALB(g/L)	23.2(19.9–27.9)	26.0(21.3–30.4)	0.007
BUN(mmol/L)	4.6(3.7–5.4)	5.3(4.0-6.7)	0.001
Scr(μmol/L)	66.7(54.5–78.8)	72.9(63.7–84.1)	0.027
UA(μmol/L)	360.0(300.5-420.5)	367.6(295.8-451.1)	0.541
CysC(mg/L)	1.1(0.94–1.3)	1.1(0.9–1.4)	0.409
eGFR(mL/min/1.73m^2^)	95.9(80.2-109.6)	100.2(90.6-108.6)	0.164
TG(mmol/L)	2.2(1.6–3.7)	2.4(1.6–3.8)	0.568
TCHO(mmol/L)	7.4(6.1–8.9)	8.2(6.3–10.2)	0.012
D-dimer(μg/L)	242.0(149.0-505.8)	240.0(150.0-542.0)	0.840
sPLA2R-Ab(RU/mL)	58.0(10.0-142.2)	59.4(27.8-121.2)	0.564
Pathologic stage(%)			0.882
Stage I	10(9.6)	7(6.9)	
Stage II	73(70.2)	73(71.6)	
Stage III	19(18.3)	21(20.6)	
Stage IV	2(1.9)	1(1.0)	
Treatment options(%)			
RAAS inhibitors	82(63.8)	72(70.6)	0.229
CTX	51(39.2)	48(47.1)	0.632
CNI	37(28.5)	24(23.5)	0.397
CD20 monoclonal antibody	2(1.5)	1(0.1)	1.000
Other immunosuppressant	1(0.8)	0(0)	1.000
Remission(%)			
CR	45(34.6)	25(24.5)	0.096
PR	44(33.8)	45(44.1)	0.110
Progression(%)			
NR	41(31.5)	32(31.4)	0.978

UWBC, uric white blood cell; HGB, hemoglobin; UA, uric acid; CysC, cystatin C; TG, triglycerides; TCHO, total cholesterol

### Feature selection

**Fig. 2:**
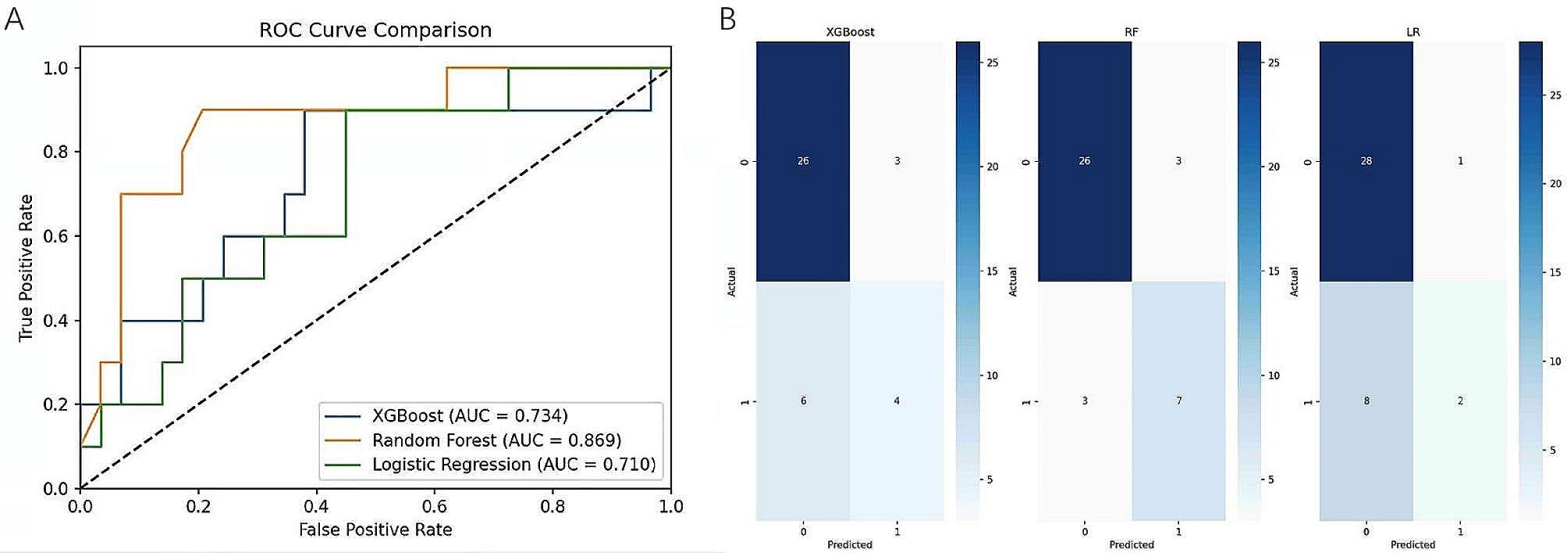
Evaluation of the predictive models. **A**: The ROC curves from three models. **B**: The confusion matrix from three models

As shown in Table 2, after the multivariable’ analysis, we identified four major risk factors: course ≥ 6 months, UTP, D-dimer, and sPLA2R-Ab. These four variables were used to construct XGBoost, RF and LR machine learning models to predict the prognosis of IMN patients. The performance evaluation results of the three models were shown in Table 3, and the ROC curve and confusion matrix were used to evaluate the model discrimination ability, as shown in Fig. 2. The performance difference between the models were significant, and the RF model had the best performance, with the highest AUROC (0.869), sensitivity (0.700), specificity (0.897), precision (0.700), accuracy (0.769) and F1-score (0.700).

**Table 2:** Variables associated with treatment response of IMN in the univariable’ and multivariable’ analyses

Variable	Univariable’ logistics regression		Multivariable’ logistics regression	
	OR (95% CI)	*P* Value	OR (95% CI)	*P* Value
Gender	2.009(0.876,4.608)	0.099		
Age	0.996(0.976,1.027)	0.805		
Weight	1.030(1.003,1.058)	0.028	1.035(1.000,1.072)	0.051
Course ≥ 6	0.370(0.168,0.818)	0.014	0.225(0.081,0.628)	0.004
History of hypertension	1.796(0.734,4.396)	0.200		
URBC	1.026(1.004,1.048)	0.019	1.031(1.000,1.063)	0.053
UWBC	1.011(0.979,1.045)	0.494		
UTP	1.172(1.083,1.268)	<0.001	1.140(1.029,1.262)	0.012
HGB	1.014(0.992,1.037)	0.213		
ALB	0.937(0.871,1.008)	0.083		
BUN	1.205(0.917,1.583)	0.182		
Scr	1.008(0.989,1.027)	0.414		
UA	1.002(0.998,1.006)	0.227		
CysC	1.376(0.328,5.773)	0.662		
eGFR	1.004(0.989,1.019)	0.628		
TG	1.209(1.017,1.438)	0.031	1.001(0.778,1.242)	0.886
TCHO	0.951(0.810,1.117)	0.543		
D-dimer	1.001(1.000,1.002)	0.006	1.001(1.000,1.002)	0.009
sPLA2R-Ab	1.005(1.002,1.009)	0.002	1.005(1.001,1.008)	0.006
RAAS inhibitors	0.515(0.229,1.158)	0.108		
Immunosuppressant therapy	0.774(0.347,1.726)	0.532		
CD20 monoclonal antibody	0.455(0.028,7.452)	0.581		

**Table 3:** Performance of the prediction models generated by the three machine learning models

Models	AUC	Sensitivity	Specificity	Precision	Accuracy	F1-score
LR	0.710	0.200	0.966	0.667	0.769	0.308
XGBoost	0.734	0.400	0.897	0.571	0.769	0.471
RF	0.869	0.700	0.897	0.700	0.846	0.700

### Model construction and comparison

**Fig. 3:**
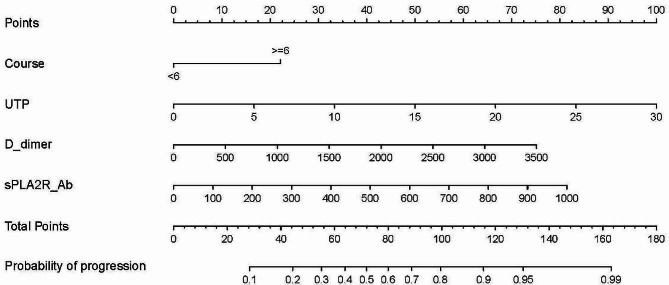
A constructed nomogram for predicting urine protein non-remission at 2 years in patients with IMN

**Fig. 4:**
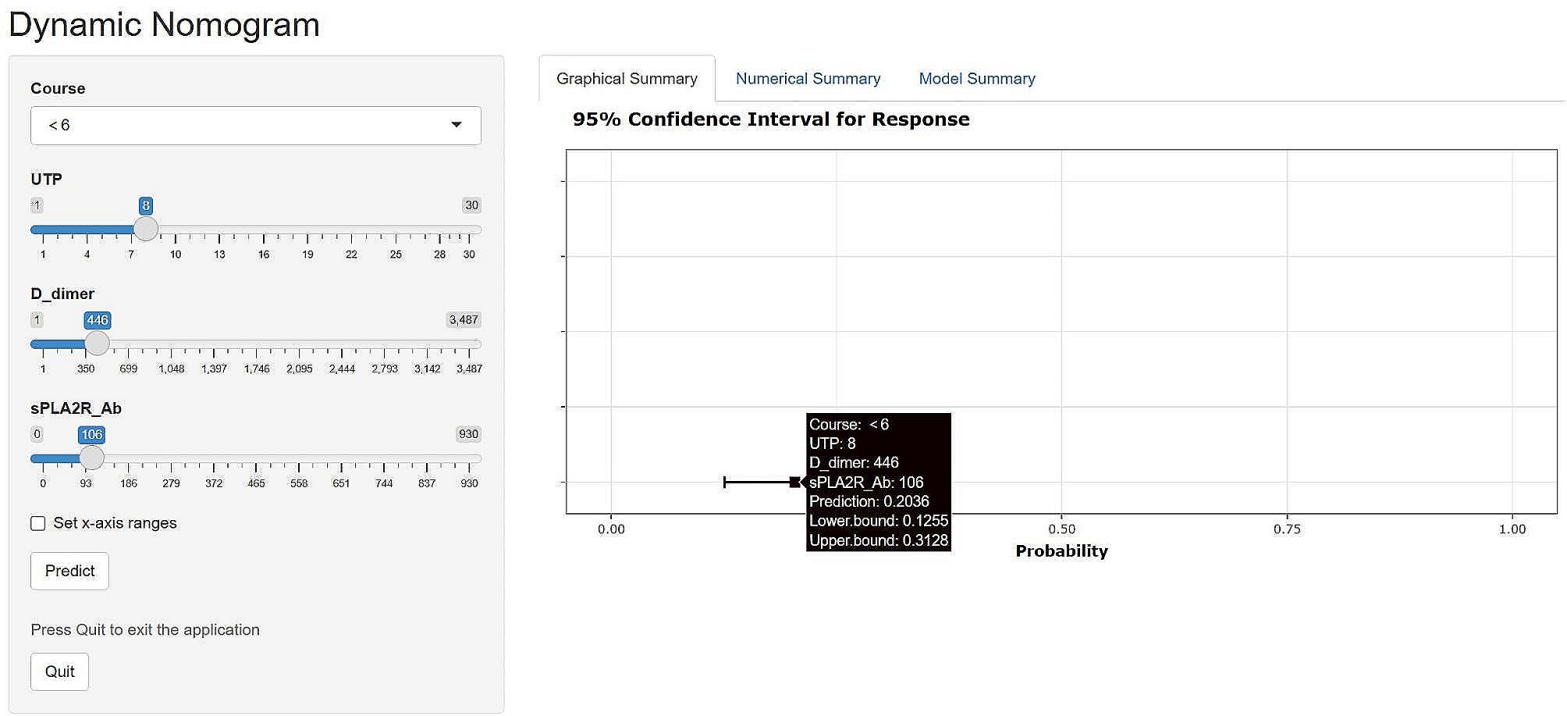
A dynamic online nomogram for predicting prognosis and response to treatment in patients with IMN. In this simulated case: the patient had a course less than 6 months, UTP 8 g/d, D-dimer 446 μg/L and sPLA2R-ab 106RU/ML, the probability of proteinuria non-remission at 2 years was 20.4%

To make the model more practical and easier to visualize, we developed a nomogram using the four predictors (course ≥ 6 months, UTP, D-dimer, and sPLA2R-Ab) (Fig. 3). For each predictive factor in the nomogram, the point was read out by drawing a line straight upward from each predictor to the point axis. The total point was calculated by summing up each point located in the total point axis, which was further converted to probability. Furthermore, a dynamic online nomogram was available via an internet interface at https://progression.shinyapps.io/DynNomapp/ (Fig. 4).

### The internal validation of the model

**Fig. 5:**
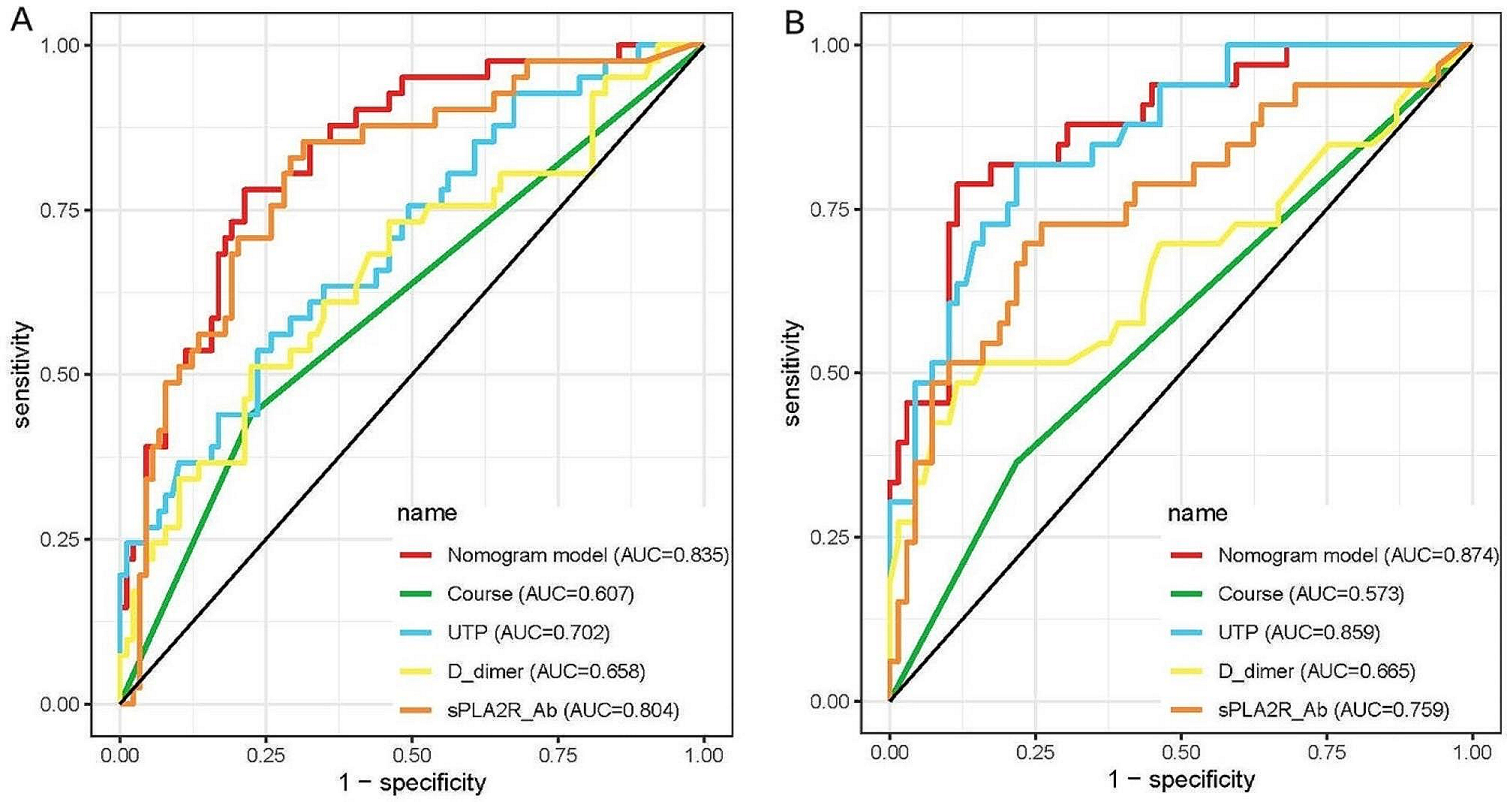
The ROC curves of the nomogram. The ability of the nomogram was measured and compared according to area under the curve values for the training (**A**) and the validation (**B**) cohorts

**Fig. 6:**
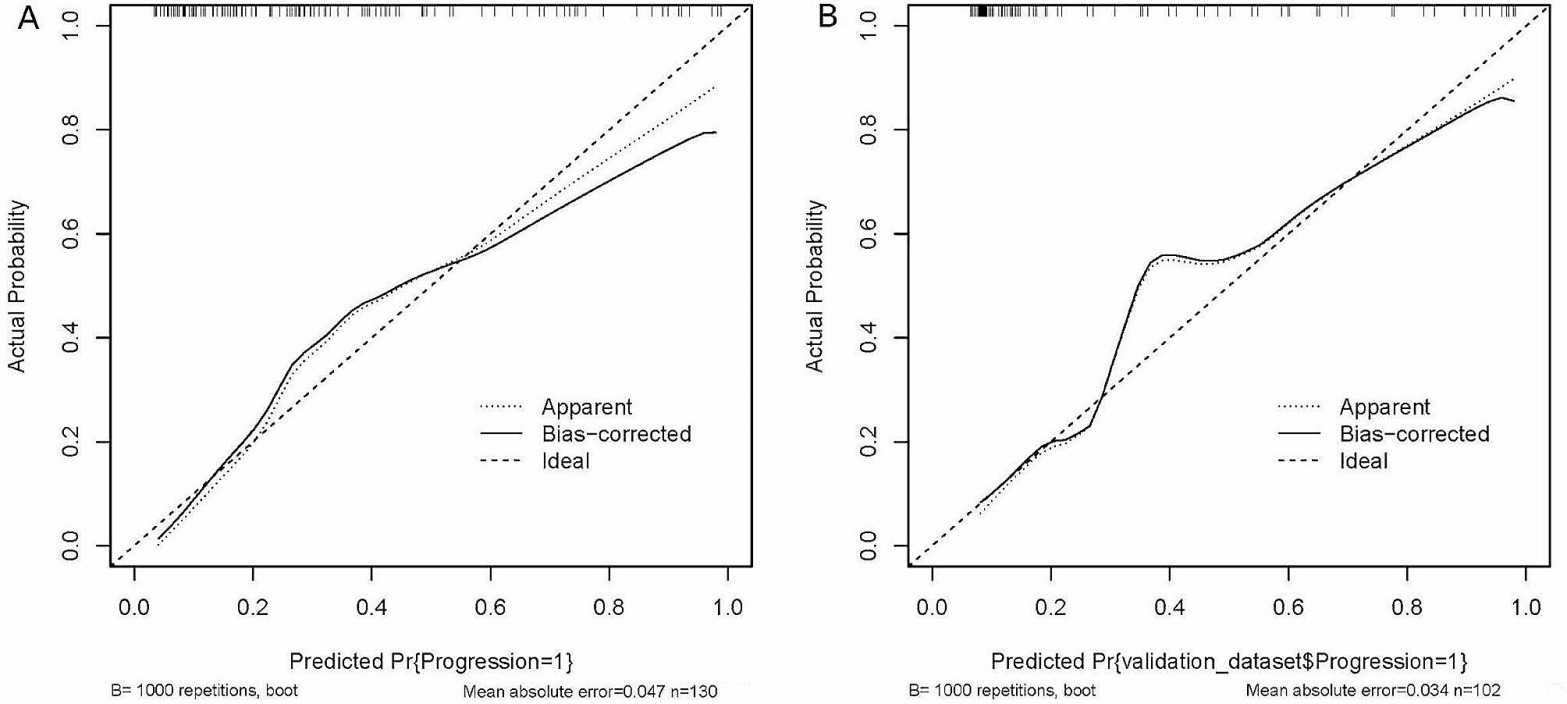
The calibration curves for the nomogram. A completely accurate prediction model will generate a plot where the probability of the actual observed and predicted corresponding completely and fall along the 45°line. The apparent calibration curve represents the calibration of the model in the development data set, while the bias-corrected curve is the calibration result after correcting the optimism with the 1000 bootstrap-resampling. The closer the apparent calibration curve is to the bias-corrected curve, the more accurately the model predicts prognosis. **A**: In the training cohort; **B**: In the validation cohort

**Fig. 7:**
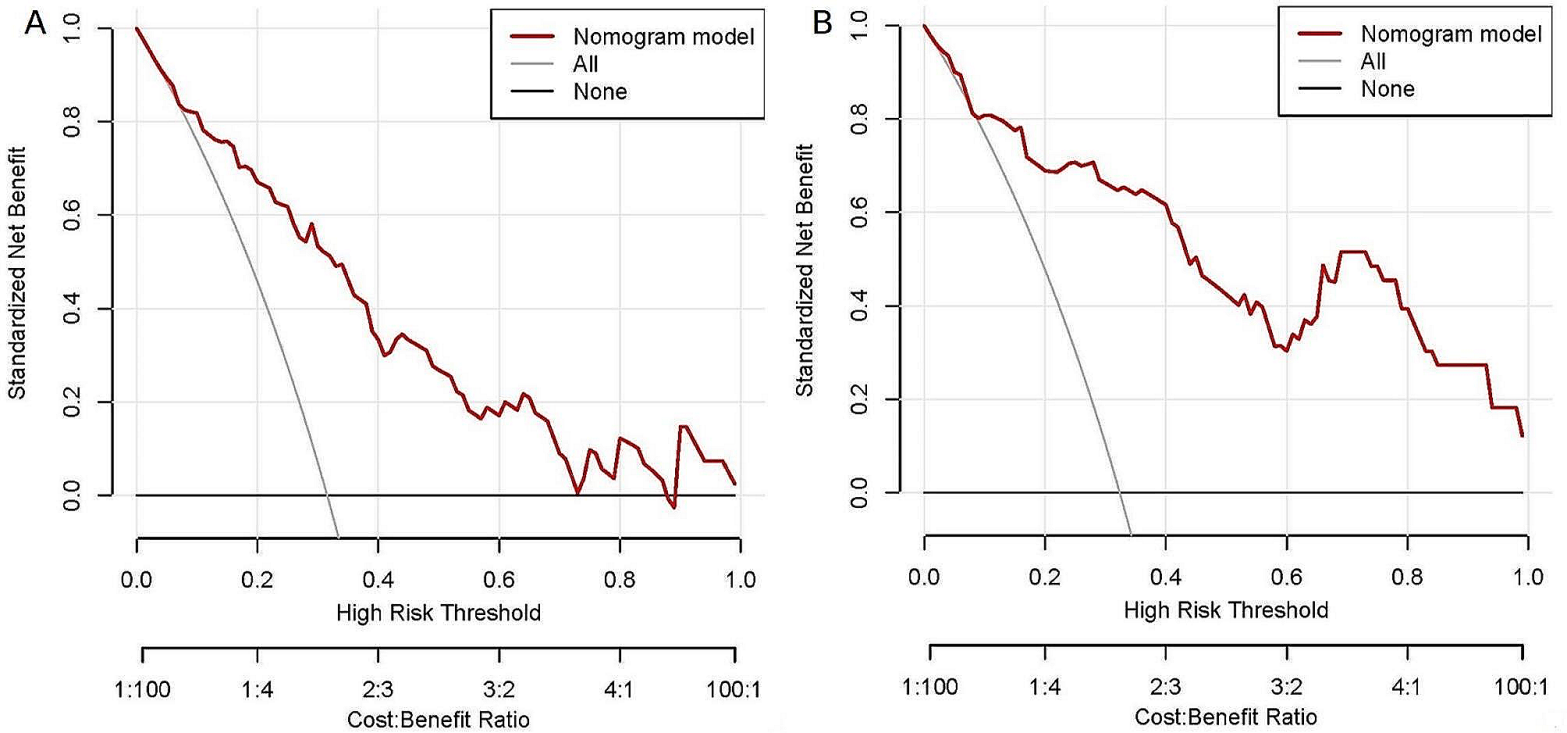
The DCA curves analysis for IMN prognosis nomogram in (**A**) the training and (**B**) the validation cohorts. The y-axis tested the net benefit. The thin gray line indicates that all patients with IMN are assumed to have non-remission of urine protein at 2 years, while the thick black line indicates that all patients with IMN are assumed to have a remission of proteinuria. The thick red line represented the risk nomogram. In training group, the decision curve showed that if the threshold probability of a patient is between 0.01 to 0.88 or greater than 0.9, using the nomogram in the present study to predict IMN prognosis adds more benefit

In the training cohort, the C-index for the nomogram was 0.835 (95% CI 0.762–0.908) and the ROC curve displayed in Fig. 5A. Z test showed that the discriminative ability of the nomogram prediction was significantly higher than that of individual predictions (course ≥ 6 months, UTP and D-dimer, Table 4). The calibration plot of the nomogram was plotted in Fig. 6A and demonstrated a good correlation between observed and predicted progression with a mean absolute error of 0.047. The DCA of the nomogram was presented in Fig. 7A, and showed that if the threshold probability of was between 10 and 88% or greater than 90%, using the nomogram to predict the IMN progression added more net benefit.

**Table 4:** Z test in the training cohort and validation cohort

Variable	Training cohort		Validation cohort	
	Z Value	*P* Value	Z Value	*P* Value
nomogram-course ≥ 6	4.491	<0.001	5.650	<0.001
nomogram-UTP	3.281	0.001	1.664	0.096
nomogram-D-dimer	3.163	0.002	3.690	<0.001
nomogram-sPLA2R-Ab	0.748	0.454	2.761	0.006

### The external validation of the nomogram

In the validation cohort, the C-index was 0.874 (95% CI 0.801–0.946, Fig. 5B) and z test showed that the nomogram discrimination was better than the individual indicator (course ≥ 6 months, D-dimer, and sPLA2R-Ab). In addition, in order to evaluate the good calibration ability of the nomogram, we also calculated other evaluation metrics beyond the AUROC based on the cut-off value and the threshold of 0.5, including the sensitivity, specificity, precision, and F1-score, and the results showed that it may be better to assess the patient’s ability to calibrate the mode based on cut-off values (Table 5). A calibration curve (Fig. 6B) also showed high consistency between predicted prognosis probability and actual prognosis proportion. The DCA curve showed that the use of the nomogram increased the net benefit and had a strong clinical utility in predicting IMN prognosis (Fig. 7B).

**Table 5:** The other evaluation metrics beyond the AUROC

Metrics	Training cohort		Validation cohort	
	Cut-off 0.296	0.5 as the threshold	Cut-off 0.330	0.5 as the threshold
sensitivity	0.780	0.512	0.781	0.438
specificity	0.787	0.921	0.886	0.914
precision	0.627	0.750	0.758	0.700
F1-score	0.695	0.609	0.769	0.539

## Discussion

As a quantitative tool for risk and benefit assessment, clinical prediction model can provide more intuitive and rational information for doctors, patients and medical policy makers. In recent years, a number of nomograms with IMN had been established [17–20], which were used to predict progression or relapse of patients with IMN, and to distinguish malignancy-associated membranous nephropathy from IMN. Compared to the above researches, the endpoint of this study was the non-remission of proteinuria at 2-year follow-up, in order to evaluate the patient’s response to treatment. Furthermore, we constructed a dynamic online nomogram model, which was multi-indexed, simple and operable, without cumbersome formulas and calculations, and the external validation also showed the universality and applicability of the model. We only needed to slide and select the value of each variable to obtain the probability of non-remission of proteinuria in the patient. The most important thing was that there was no manual intervention in the whole process, which avoided accidental errors.

According to current reports, this is the first dynamic online nomogram based on baseline parameters to predict treatment response in patients with IMN. The nomogram has been validated internally and externally to show that it has good discrimination, calibration ability and clinical net benefit. And based on the nomogram, the clinician can preliminarily judge the patient’s prognosis and response to treatment after 2 years, fully communicate with the patient, and choose the most suitable personalized treatment plan for the patient.

The results of the retrospective study indicated that course ≥ 6 months, UTP, D-dimer, and sPLA2R-Ab were significant independent predictors of poor response in patients with IMN. What makes our study unique is that it links D-dimer, a marker of thromboembolic complications in IMN, to prognosis and confirms that D-dimer is an independent risk factor for urine protein remission in IMN. D-dimer is a specific product of cross-linked fibrin under the action of plasmin [26], which can be used as an important molecular marker to reflect the plasma hypercoagulability state and the activation of the fibrinolytic system in vivo [27, 28]. IMN is an immune-mediated inflammatory disease with a high risk of thromboembolic complications due to damage to vascular endothelial cells, activation of the coagulation system, and weakening of the fibrinolytic system [29, 30]. Persistent proteinuria in patients with IMN presenting with nephrotic syndrome may lead to secondary venous thrombosis, increasing the risk of infection and acute kidney injury, and thus leading to poor prognosis in patients with IMN [29, 31]. Therefore, the IMN patients with high levels of D-dimer may indicate a high risk of thrombotic events and critical condition in IMN patients, and need to initiate anticoagulation and immunosuppressive therapy as soon as possible [32, 33].

IMN is a slowly progressive immune and inflammation-associated renal disease [34]. We also found that patients with a long course of disease had a poor response to treatment, which may be due to the persistence of chronic inflammation, resulting in increased deposition of immune complexes on the outside of the glomerular basement membrane, massive formation of basement membrane “spike” and thickening of the basement membrane, thereby aggravating renal injury and leading to poor prognosis [35, 36]. Moreover, studies had shown that immune-inflammation index and monocyte-lymphocyte ratio were reliable markers which might be used to predict prognosis for IMN patients [37, 38].

Previous studies and well-known researchers agree that the prognosis of IMN patients is closely related to UTP and sPLA2R-Ab levels. The heavier UTP and the higher sPLA2R-Ab level, the worse the prognosis for patients with IMN. Higher proteinuria level is significantly associated with a higher risk of reduction in renal function [39, 40]. Persistent proteinuria is an independent risk factor for progression of IMN to ESRD. The results of the present study cohort indicated that the 24-h proteinuria level was an independent predictor for a poor renal outcome, which was consistent with the present reports.

It is well documented that PLA2R and its autoantibodies are closely related to the prognosis of IMN [41–43]. Compared with glomerular PLA2R deposition, serum anti-PLA2R antibody levels are more closely correlated with renal outcome [44]. The KDIGO 2021 glomerular disease management guidelines recommend longitudinal monitoring of sPLA2R-Ab levels at 6 months after start of treatment may be useful for evaluating treatment response in patients with MN, and can be used to adjust the treatment strategy [14]. Consistent with current findings, we also confirmed a significant association between baseline sPLA2R-Ab levels and renal outcome in IMN patients.

Unfortunately, we did not find satisfactory results for common prognostic markers of IMN, such as serum albumin [14, 45]. First, the nomogram predicts that most patients with non-remission will have refractory MN and will endpoint with the time outcome, while previous studies have mostly ended with event outcomes, which may lead us to different results. And secondly, we suspect that this may be due to the liver’s strong ability to synthesize albumin, and of course there is some correlation with our small sample size, and we hope to conduct further studies on large sample sizes to illustrate the association between them. Furthermore, our findings did not find that treatment regimens had a significant effect on urine protein outcomes. However, RAAS inhibitors, immunosuppressant, and CD20 monoclonal antibody therapy all showed low odds ratios in univariable’ logistics regression, implying that certain patients might benefit from these treatments. Therefore, based on our model, we recommend that when assessing the outcome of patients with urine protein, if the probability of non-remission of urine protein is high and the likelihood of disease progression is high, we should actively communicate with the patient and take intervention to achieve a good prognosis.

The present study developed a dynamic online nomogram model for the early prediction of poor treatment response in IMN patients, and we can formulate individualized treatment and management plans, and determine whether it is appropriate to initiate immunosuppressive therapy to reduce the risk of progression to ESRD. But, there are several limitations to the present study. First, this study covered data from three study centers, the failure to establish a unified testing platform resulted in differences in validation and training cohorts baseline data. Conversely, it also verified the universality and applicability of the prognostic model. Second, recent studies showed that chronic tubulointerstitial inflammation was considered as a risk factor for poor renal prognosis in patients with IMN [19, 46]. This retrospective study had a small sample size, and the urinary α1/β2-microglobulin were not been included the association between chronic tubulointerstitial inflammation and poor prognosis of IMN had not been studied. Therefore, there is a need for a prospective, multicenter, large- scale cohort to explore this correlation.

## Conclusions

In conclusion, we developed a dynamic online model for assessing patient prognosis and treatment response in patients with IMN and validated the model using independent patient cohorts. The nomogram is easy to use and can identify patients with IMN who are at high risk of poor response to treatment and a poor prognosis, and may help clinicians formulate an individualized treatment plan for patients and discuss when to start immunosuppressive therapy for a good prognosis.

## Data Availability

The data used to support the findings of this study are available from the corresponding author upon request.

## Abbreviations

ACEi: Angiotensin-converting enzyme inhibitor
ALB: Albumin
ARB: Angiotensin II receptor blocker
AUROC: Area under the receiver operating characteristic
BUN: Blood urea nitrogen
CI: Confidence interval
C-index: Concordance index
CNI: Calcineurin inhibitor
CR: Complete remission
CTX: Cyclophosphamide
Cysc: Cystatin C
DCA: Decision curve analysis
eGFR: Estimate glomerular filtration rate
ESRD: End stage renal disease
HGB: Hemoglobin
IMN: Idiopathic membranous nephropathy
IQR: Interquartile ranges
KDIGO: Kidney Disease Improving Global Outcomes
LR: Logistic regression
NR: non-remission
NS: Nephrotic syndrome
PR: Partial remission
RF: Random forest
RAAS: Renin-angiotensin-aldosterone system
Scr: Serum creatinine
SMN: Secondary membranous nephropathy
sPLA2R-Ab: Serum anti-phospholipase A2 receptor antibody
TCHO: Total cholesterol
TG: Triglycerides
UA: Uric acid
URBC: Uric red blood cell
UTP: 24 h urinary protein
UWBC: Uric white blood cell
XGBoost: Extreme gradient boost
